# Using reinforcement learning in genome assembly: in-depth analysis of a Q-learning assembler

**DOI:** 10.3389/fbinf.2025.1633623

**Published:** 2025-08-20

**Authors:** Kleber Padovani, Rafael Cabral Borges, Roberto Xavier, André Carlos Carvalho, Anna Reali, Annie Chateau, Ronnie Alves

**Affiliations:** ^1^ Center for Higher Studies of Itacoatiara, University of the State of Amazonas, Itacoatiara, Amazonas, Brazil; ^2^ Data Science, Vale Institute of Technology, Belém, Pará, Brazil; ^3^ Institute of Mathematics and Computer Sciences, University of São Paulo, São Carlos, São Paulo, Brazil; ^4^ Polytechnic School, University of São Paulo, São Paulo, Brazil; ^5^ Laboratory of Computer Science, Robotics and Microelectronics of Montpellier, University of Montpellier, Montpellier, France; ^6^ Institute of Natural Sciences, Federal University of Pará, Belém, Pará, Brazil

**Keywords:** reinforcement learning, genome assembly, machine learning, artificial intelligence, bioinformatics, q-learning

## Abstract

Genome assembly remains an unsolved problem, and de novo strategies (i.e., those run without a reference) are relevant but computationally complex tasks in genomics. Although de novo assemblers have been previously successfully applied in genomic projects, there is still no “best assembler”, and the choice and setup of assemblers still rely on bioinformatics experts. Thus, as with other computationally complex problems, machine learning has emerged as an alternative (or complementary) way to develop accurate, fast and autonomous assemblers. Reinforcement learning has proven promising for solving complex activities without supervision, such as games, and there is a pressing need to understand the limits of this approach to “real-life” problems, such as the DNA fragment assembly problem. In this study, we analyze the boundaries of applying machine learning via reinforcement learning (RL) for genome assembly. We expand upon the previous approach found in the literature to solve this problem by carefully exploring the learning aspects of the proposed intelligent agent, which uses the Q-learning algorithm. We improved the reward system and optimized the exploration of the state space based on pruning and in collaboration with evolutionary computing (>300% improvement). We tested the new approaches on 23 environments. Our results suggest the unsatisfactory performance of the approaches, both in terms of assembly quality and execution time, providing strong evidence for the poor scalability of the studied reinforcement learning approaches to the genome assembly problem. Finally, we discuss the existing proposal, complemented by attempts at improvement that also proved insufficient. In doing so, we contribute to the scientific community by offering a clear mapping of the limitations and challenges that should be taken into account in future attempts to apply reinforcement learning to genome assembly.

## 1 Introduction

The genome of an organism is the sequence of all nucleotides from its DNA molecules. Each isolated nucleotide represents no relevant biological information, and within the organism’s genome, there are species genes that define species traits and behaviors (e.g., eye color) (Portin and Wilkins, 2017). A single DNA fragment cannot represent the complete information from a gene, and genome assembly is the computational task used to order the sequenced DNA fragments (i.e., *reads*) and reconstruct the original DNA sequence (Heather and Chain, 2016). The size and number of *reads* directly influence the complexity of the assembly process, and illuminating this bottleneck problem has become an important bioinformatics problem for producing a fast, automated and reliable solution.

Genome assemblers adopt comparative and/or *de novo* strategies. A comparative strategy requires a reference genome (Ji et al., 2017). *De novo* strategies (i.e., do not need a reference) are particularly important given that only a small number of reference genomes are currently available (Wong et al., 2020). However, this approach is a highly complex combinatorial problem that falls into the theoretically intractable class of computational problems (NP-hard) (Medvedev et al., 2007). *De novo* assemblers (commonly based on heuristics and graphs) can produce acceptable solutions but require specific bioinformatics knowledge for configuration and parameter setting, and optimal results are not guaranteed (Gurevich et al., 2013).

Machine learning has emerged as a powerful alternative for addressing computationally complex problems (Jamialahmadi et al., 2024), where finding exact solutions is often computationally infeasible. Instead of exhaustively exploring all possibilities, machine learning models can learn patterns from data to provide approximate yet acceptable solutions within reasonable time constraints (Souza et al., 2018). This approach has gained particular relevance in genomics and related fields, where complex computational problems, such as drug repositioning (Zhao et al., 2025; Zhao et al., 2022), gene prediction (Silva et al., 2021), designing of antimicrobial peptides (Wang et al., 2025a), assembling 3D molecular structures (Wang et al., 2025b), pose significant computational challenges.

The genome assembly problem is NP-hard because the Shortest Common Superstring Problem, which is NP-hard, can be polynomially reduced to the genome assembly problem (Fernandez et al., 2024). This means that solving the genome assembly optimally is at least as hard as solving the Shortest Common Superstring Problem. The complexity arises from the need to find an ordering of reads that minimizes the total assembled sequence length, which involves searching through an exponentially large combinatorial space, making exact solutions computationally infeasible for real-world genome sizes (Souza et al., 2018).

Genome assembly is currently not a fully solved problem. However, few approaches have applied machine learning to achieve better solutions for the assembly problem (Souza et al., 2018; Yassine and Riffi, 2023). With the current availability of increased processing and storage power, machine learning applications have grown significantly, and notable results have been reported (LeCun, 2019). This increase also enabled the resurgence of reinforcement learning applications to address these problems (Botvinick et al., 2019).

Reinforcement learning (RL) is a basic machine learning paradigm in which intelligent agents take action in a task environment. Ideally, this agent solves the task when it is able to learn how to maximize the rewards from its actions (Sutton and Barto, 2018). Despite RL’s impressive achievements in games, especially those leveraging deep reinforcement learning, translating this success to real-world problems remains a significant challenge (Dulac-Arnold et al., 2019; Crespo and Wichert, 2020; Osborne et al., 2022). The limited adoption of RL in real-world applications is also evident in the specific case of genome assembly, with only a few studies identified (Souza et al., 2018; Bocicor et al., 2011a; Xavier et al., 2020; Karami et al., 2023).

A brief literature review revealed few studies applying reinforcement learning (RL) to the fragment assembly problem, identifying only three attempts (for details, see Section 7 of the Supplementary Material (Padovani and Alves, et al., 2020)). The first approach, which serves as the foundation for the present study, applies Q-learning with a reward system based on overlap scores, where each action adds an assembled read, yielding optimal results on small datasets (Bocicor et al., 2011a). The second uses distributed collaborative agents to address convergence and state space issues, improving the execution time but still facing scalability limitations (Bocicor et al., 2011b). The third evaluated the scalability of (Bocicor et al., 2011a) and, despite persistent complexity challenges, achieved remarkable results on small-to medium-sized datasets (Xavier et al., 2020).

In this context, investigating both the limitations and the potential of this approach is particularly relevant, as it remains innovative despite its conceptual simplicity. This study aims to critically analyze one of the few attempts to apply RL to fragment assembly—hereafter referred to as the seminal approach—evaluating its scalability, performance, and generalizability through new experiments involving greater complexity and larger data volumes. Furthermore, it proposes enhancements to the original strategy, aiming to overcome identified limitations and further explore the practical potential of reinforcement learning in this domain.

This investigation is motivated both by the originality of this research direction and by the need to understand the extent to which RL can offer practical advantages over established methods when applied to the assembly problem, thus contributing to the expansion of scientific knowledge and guiding future research at the intersection of bioinformatics and artificial intelligence (Jamialahmadi et al., 2024).

## 2 Materials and methods

The seminal approach (Bocicor et al., 2011a) proposes an episodic trained agent (whose training has been divided into episodes) applying the Q-learning reinforcement learning algorithm, which allows the agent to learn through the consequences (positive or negative rewards) received after taking action. The ability to obtain intelligent and trained agents via RL using the seminal approach is important because it could eliminate the need for human specialists.

The Q-learning algorithm requires a Markov decision process definition with established parameters of states and actions, together with a reward system to be achieved by the agent at each action in every state (Sutton and Barto, 2018). The problem was then modeled through a state space capable of representing all possible read arrangements with repetition, with one action for each read in each state (Bocicor et al., 2011a). Following these definitions, from graph theory, the proposed state space for n *reads* can be visualized as a complete *n*-ary tree, with a height equal to *n*, as the set of states presents one initial state and forms a connected and acyclic graph (Cormen et al., 2009). The number of existing states in the state space is represented by Equation 1.

$$number\;of\;states=\frac{n^{n+1}-1}{n-1}$$ (1)

**FIGURE 1:**
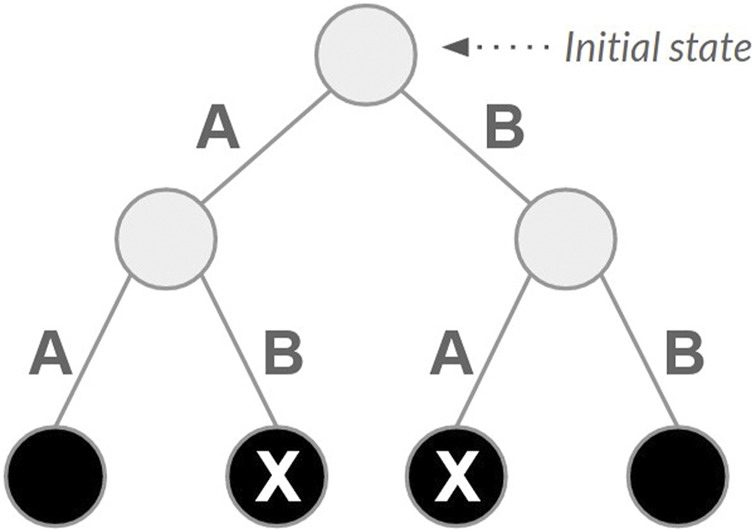
Example of a state space for a set of two reads, referred to here as A and B.

The reward system depends on the type of state reached after each action (absorbing or nonabsorbing). An absorbing state is one that, once entered, cannot be left; it has no outgoing transitions to other states (Sutton and Barto, 2018; Grinstead and Snell, 2012). Each state requiring *n* actions to be reached (with n being the number of *reads*) is an absorbing state. A small and constant reward (i.e., 0.1) is assigned for each action. Finally, actions leading to other absorbing states produce a reward corresponding to the sum of overlaps between all pairs of consecutive *reads* used to reach these states. Figure 1 presents a state space example for a set of 2 *reads* (A and B) with a single initial state, two actions associated with nonabsorbing states and four absorbing states (highlighted black circles), achieved after taking two actions.

Two absorbing states are highlighted (X). These are the final states, as they are reached directly without repeated actions.

The Smith‒Waterman algorithm (SW) was applied to obtain the overlaps between pairs of *reads* and added to obtain the rewards of actions leading to the final states (Smith and Waterman, 1981). The sum of overlaps when reaching a final state *s* (Performance Measure - PM) is described in Equation 2, where *reads* correspond to the sequence of *reads* associated with the actions for achieving *s*. In an optimal solution, repeated *reads* overlap completely, and pairs reach the maximum PM.

$$PM\left(s\right)=\sum_{i=1}^{n-1}sw\left({read}_{s}\left[i\right],{read}_{s}\;\left[i+1\right]\right)$$ (2)

Based on these definitions, the seminal approach produced positive results against two sets of 4 and 10 simulated *reads* less than 10 *base pairs (bp)* and 8 *bp,* respectively. A scalability analysis was applied to evaluate the performance of this approach against 18 datasets produced following the same simulation methods (Xavier et al., 2020). The initial set is one of the sets featured in the seminal approach, containing 10 *reads* with 8 *bp* extracted from a 25 *bp* microgenome. Seventeen new datasets were generated from this microgenome and from a novel 50 *bp* microgenome (8 from the minor microgenome and 9 from the major microgenome), each containing 10, 20 or 30 *reads*, with 8 *bp*, 10 *bp* or 15 *bp*.

All the previous definitions were replicated, but *α* and *ɣ* were set to 0.8 and 0.9, respectively. The former controls how much newly learned values (accumulated rewards) influence the update of the Q-table—the closer to 1, the greater the influence—and the latter controls how much the agent values estimated future rewards compared with immediate ones—the closer to 1, the greater the influence (i.e., the less impulsive the agent becomes). With the chosen values, the agent theoretically learns quickly from new experiences (new sequences of reads) while still valuing potential future rewards, which is suitable for scenarios with sparse rewards and high payoffs concentrated at the end of episodes.

The space of actions was reduced so that actions associated with previously taken *reads* were removed from the available actions (Xavier et al., 2020). In the state space depicted in Figure 1, the leftmost and rightmost leaves (i.e., absorbing states) are removed after this change. The number of states decreases because the tree has n nodes at height 1, n (n−1) nodes at height 2, n (n−1) (n−2) nodes at height 3, and so on. Assuming that i corresponds to the height and ranges from 0 to n, we can represent these quantities using the summation given in Equation 3. Although the number of states is reduced, the size of the state space still grows exponentially.

$$number\;of\;states=\sum_{i=0}^{n}\frac{n!}{\left(n-i\right)!}$$ (3)

This confirmed positive results from the seminal approach with the first dataset; however, the performance decreased with increasing size, reaching the target microgenome in only 2 out of the 17 major datasets. This may be related to the high cost required by the agent to explore a vast state space and to failures in the reward system (Xavier et al., 2020). Thus, to investigate the application of reinforcement learning to genome assembly and address the current challenge of applying RL to real-world problems (Dulac-Arnold et al., 2019), in this study, we analyzed the limits of RL to the Genome Assembly problem, a key problem for scientific development. We corrected previously described issues, explored the performance of an improved reward system and added complementary strategies to be incorporated into the seminal approach to obtain improved and automated genome assemblies through machine learning applications.

**FIGURE 2:**
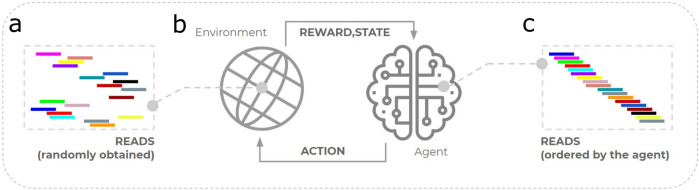
Illustration of the application of reinforcement learning to the genome assembly problem. **(a)** The set of *reads* is represented computationally by a reinforcement learning environment. **(b)** Successive interactions with the environment caused by taking action. **(c)** The agent ideally learns the correct order of *reads,* i.e., reaching the target genome.

In this study, 7 experiments were evaluated against the seminal approach. The experiments were implemented in Python 3.8 using the NumPy package (Harris et al., 2020). The main goal was to reach an RL-trained agent to correctly identify the order of *reads* from a sequenced genome. Figure 2 illustrates this proposal, where the environment represents the set of *reads* to assemble. The agent interacts with the environment by taking actions intended to order the *reads*. For each action taken, the environment is updated and provides a corresponding reward to the agent. The agent learns from the reward received and takes new action until reaching (ideally) the correct order of *reads*. The approaches produced here consider scalability analysis (Xavier et al., 2020), with improvements made to the reward system—especially in Approaches 1 — and to optimize the agent’s exploration—approaches 2 and 3.

### 2.1 Approaches 1: tackling sparse rewards

Approaches 1.1, 1.2, 1.3, and 1.4 aimed at improving the reward system, as given by Equation 4. Optimally, the agent achieves the correct order of *reads* upon learning the set of actions, specifically a permutation of *reads* that maximizes the accumulated reward. Thus, the optimal actions (those leading to the anticipated permutation of reads) must always yield the highest cumulative reward. Nevertheless, this proposition may not hold consistently for the reward system proposed in the seminal approach, as it allows some nonoptimal actions to result in maximum accumulated rewards.

$$r\left(s,a,s^{\prime }\right)=\left\{\begin{array}{l} PM\left(s^{\prime }\right)\;if\;s^{\prime }\;is\;a\;final\;state,\\ 0.1\;otherwise \end{array}\right.$$ (4)

This inconsistency (details in Section 6 of the Supplementary Material (Padovani and Alves, 2020)) stems from the sequence alignment with the Smith‒Waterman algorithm (SW), which calculates a score to represent major alignment size (even if partial) but has no constraint on the order between sequences. Thus, the overlap score from the SW might induce the agent to find *read* permutations with high overlap values in pairs of *reads* without any suffix-prefix alignment. Therefore, using the PM score as a reward for training may be ineffective for some datasets.

Thus, to improve the agent’s performance, we adjust the reward system through four approaches to explore two aspects: (a) the use of an overlap score that considers the relative order of *reads* and/or (b) the use of dense rewards. These new reward systems are presented in approaches 1.1, 1.2, 1.3 and 1.4.

As in the seminal approach, approach 1.1 defines that actions leading to the final states produce a bonus reward (of 1.0), added to another numerical overlap score between all subsequent *reads* used since the initial state. Thus, a reward corresponding to the sum of the normalized overlap score (ranging from 0 to 1) of each pair of *reads* was produced considering their relative order. Every action leading to nonfinal states produces constant and low rewards (0.1). Equation 5 formalizes the reward system for Approach 1.1, with *PM*_*norm*_ (*s′*) representing the normalized overlap between the *reads* used to reach *s′* (details in Section 2 of the Supplementary Material (Padovani and Alves, et al., 2020)).

$$r\left(s,a,s^{\prime }\right)=\left\{\begin{array}{l} {PM}_{\mathrm{norm}}\left(s^{\prime }\right)+1.0\;if\;s^{\prime }\;is\;a\;final\;state,\\ 0.1\;otherwise \end{array}\right.$$ (5)

Despite the overlap score considering the order of *reads* in approach 1.1, it is susceptible to the sparse rewards problem, as in the seminal approach. Although it often produces small, constant and positive rewards and not a zero-value reward, as applied in sparse reward systems, only few and sparse state‒action pairs produce higher rewards. In both systems (Equations 4, 5) no reward is provided during the learning process (since any *read* incorporated would produce a reward of 0.1).

Thus, the agent’s learning process depends exclusively on the sparse actions taken during the exploration of this state space, tending to take a long time because of the sparse reward problem (Trott et al., 2019). In approaches 1.2, 1.3 and 1.4, we focused on improving it with higher rewards distributed for each action taken in each episode (previously obtained only at the end of the episode). These approaches focus on reducing or eliminating inconsistencies that allow permutations of unaligned reads to produce maximum accumulated rewards. Equations 6–8 represent the reward systems for approaches 1.2, 1.3 and 1.4, respectively, where *ol*_*norm*_ *(s, s′)* represents the normalized overlap between two subsequent reads (which represents the ratio between the overlap length of the two reads and the maximum length of the target genome—the ratio between the overlap length of the two reads and the estimated maximum genome size, calculated as the number of reads multiplied by the read length—and *PM*_*norm*_(s) corresponds to the sum of the normalized overlaps of all the reads involved.

$$r\left(s,a,s^{\prime }\right)={PM}_{\mathrm{norm}}\left(s^{\prime }\right)$$ (6)

$$r\left(s,a,s^{\prime }\right)=\left\{\begin{array}{l} {PM}_{norm}\left(s^{\prime }\right)+1.0\;if\;s^{\prime }is\;a\;final\;state,\\ {ol}_{norm}\left(s,s^{\prime }\right)\;otherwise \end{array}\right.$$ (7)

$$r\left(s,a,s^{\prime }\right)=\left\{\begin{array}{l} {ol}_{norm}\left({s,s}^{\prime }\right)+1.0\;if\;s^{\prime }is\;a\;final\;state,\\ {ol}_{norm}\left(s,s^{\prime }\right)\;otherwise \end{array}\right.$$ (8)

### 2.2 Approach 2: pruning-based elimination action

**FIGURE 3:**
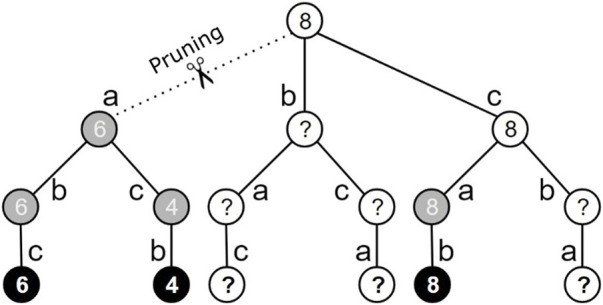
Illustration of the pruning procedure for a state space of 16 states referring to the assembly of 3 reads, referred to as a, b and c. Final states are represented by the leaf nodes of the tree. The black states correspond to final states that have already been visited. The gray states represent nonfinal states whose children have all been visited. White states (whether final or not) are nodes that have not yet been visited. The generic pruning procedure is defined in detail in Algorithm 1.

To reduce the state space from the seminal approach, a heuristic procedure was applied to eliminate fully explored actions where the maximum cumulative reward achieved was smaller than the cumulative reward from taking any other action available. In Figure 3, looking at the changed state space as a tree—removing actions associated with used reads, we see 16 states, 6 are absorbing states and the final states (tree base). Note that 3 out of the 6 final states are highlighted in black, whereas the remaining states are highlighted in gray and white. The black states correspond to the explored final states (i.e., visited by the agent). Gray states, such as those reached by taking action *a* in the initial state, represent states where all children were fully visited during the learning process. White states (final or not) are those not yet explored and/or that have unexplored children, e.g., the initial state, where one child is not explored and the other one is partially explored.

When an unexplored final state is reached, such as the rightmost final state in Figure 3, the accumulated rewards are maintained and propagated for its predecessors, maintaining only the highest value propagated for the children. Each reward is represented by integer numbers within the states in the figure. In each nonfinal state, the highest accumulated reward achieved during the training process is stored. Thus, it is possible to prune irrelevant actions that do not produce the maximum accumulated reward (e.g., action a of the initial state in Figure 3).

Note that all possible achievable states after taking this action were explored and the maximum cumulative reward was 6, whereas the initial state of action *c* alone produces a reward equal to 8. When the agent first goes through the sequence of states corresponding to actions *c*, *a* and *b*, the pruning mechanism propagates the maximum reward value up to the initial state and, at that moment, cuts action *a* from the initial state. The pseudocode presented in Algorithm 1 presents the procedure for updating the pruning process when the last explored final state (*state*) is reached, obtaining the corresponding accumulated reward achieved (*newReward*) (Table 1).

**TABLE 1:** Description of the code used for building the pruning algorithm.

Algorithm 1 Pruning’s algorithm
1: procedure Prune (state: treeNode, newReward: float) 2: if *state* ≠ *null* and (*state.unseen* or *newReward* > *state.maxReward*) then 3: *state.unseen* ← *false* 4: *state.maxReward* ← *newReward* 5: if *state.final* **then** ▷ prune children where *maxReward* < *newReward* 6: PruneUselessChildren (*state*) 7: end if 8: Prune (*state.parent*, *newReward*) 9: end if 10: end procedure

### 2.3 Approaches 3: evolutionary-based exploration

In these approaches, we explore the potential for mutual collaboration between reinforcement learning and evolutionary computing by applying the elitist selection of the genetic algorithm (Baluja and Caruana, 1995; Konar, 2005) to optimize the exploration of the state space. The individual contributions of the genetic algorithm used in this hybrid proposal were divided into two approaches: 3.1 and 3.2.

### 2.4 Approach 3.1: evolutionary-aided reinforcement learning assembly

**FIGURE 4:**
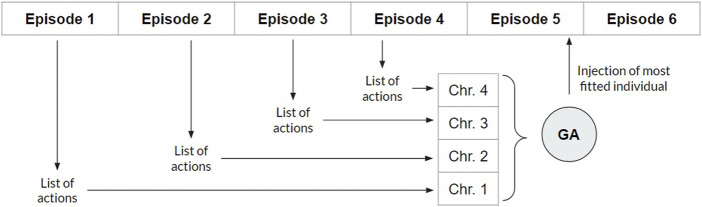
Illustration of the proposed interaction between reinforcement learning (RL) and the genetic algorithm. At each RL episode, the actions taken by the agent are converted into the chromosome (each action as a gene) of an individual of the initial population of the genetic algorithm, whose size n is predefined. After n episodes (n individuals in the initial population), this population evolves for a predefined number of generations through the genetic algorithm. Then, the most adapted individual of the last generation is obtained. In the end, that individual’s chromosomal genes are used as actions in the next RL episode.

Applying the ϵ-greedy to expand the exploration of agents trained by the Q-learning algorithm allows broader initial exploration, achieving the optimal policy once the state space has been sufficiently explored (Botvinick et al., 2019). However, the existing trade-off between exploitation and exploration remains a major problem for RL in high-dimensional environments (Gimelfarb et al., 2020; Peterson and Verstynen, 2019). Here, for the first time, we introduce the interaction between RL and evolutionary computing into the exploration process based on the operation of the Q-learning algorithm. In each episode, the sequence of actions is stored, and at the end of the episode, the sequence is transformed into a chromosome of an initial population that evolves (see Figure 4).

New chromosomes are inserted until the number of chromosomes reaches the predefined population size. At this point, agent training is interrupted, and m genetic generations are carried out—with m being predefined (see Section 4 of the Supplementary Material (Padovani and Alves, et al., 2020)) and applying the normalized sum of overlaps between reads as the adaptive function—the same as that applied in Equation 8 and detailed in Section 2 of the Supplementary Material.

**FIGURE 5:**
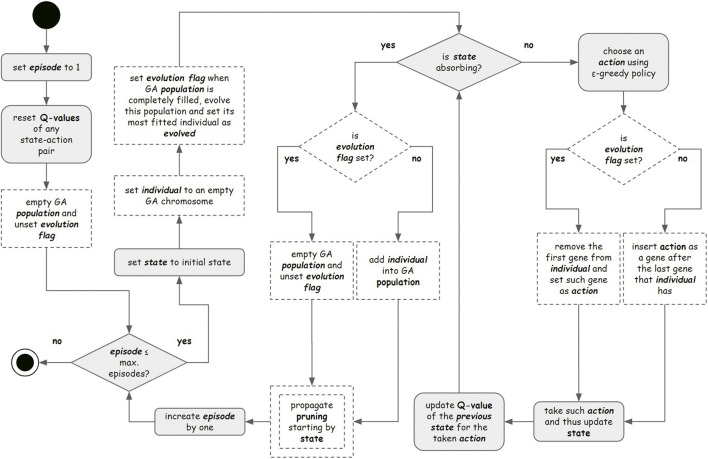
Flowchart representing all the approaches performed in this study. The gray elements represent steps performed by approaches 1.1, 1.2, 1.3, and 1.4 (reward systems analysis). The double-edged dashed element represents steps performed by approach 2 (space state pruning). Single border dashed elements represent steps performed by Approach 3.1 (hybrid approach applying the genetic algorithm - GA).

After m generations (objective function), the most fit individual is used for conducting the next episode in the agent’s RL training, hitherto interrupted. As each gene of the individual’s chromosome corresponds to one possible action, the complete gene sequence will contain distinct successive actions to be taken by the agent in the current episode, producing a mutual collaboration between RL and the genetic algorithm—the initial populations of the genetic algorithm are produced by RL and, as a counterpart, the results from the evolution of the genetic algorithm are introduced in an RL episode (Figure 5).

### 2.5 Approach 3.2: evolutionary-based assembly

To estimate the genetic algorithm contribution in Approach 3.1, its assembling performance was evaluated separately, following the same configurations set for the previous approach, but adopting as a starting population a set of individuals whose chromosomes were built from random permutations without repetition of reads.

### 2.6 Datasets and analysis

To assess the performance of all the approaches (including the seminal approach), in addition to the 18 datasets from Xavier et al., 2020, 5 novel datasets derived from microgenomes extracted in previous studies (Bocicor et al., 2011a; Xavier et al., 2020) were created. These are not arbitrary genome fragments, as were the case for previously used microgenomes (which had 25 bp and 50 bp), but represent larger fragments of previously annotated genes from the corresponding organism (i.e., *E. coli*). Given that the datasets are simulated data, no cycles in the genome were considered, which is a limitation of the approach.

The experiments were carried out with 23 datasets, detailed in Table 2—the last 5 lines are gene derived.

**TABLE 2:** Datasets used in the experiments. The first column shows the size (in bp) of the microgenome used to generate the reads of each set; the second column shows the number of reads generated; the third column shows the size of the generated reads; and the fourth column shows the name of the environment built for each set in the OpenAI Gym toolkit.

μgen. Size	# reads	read Size	Gym environment name
25	10	8	GymnomeAssembly_25_10_8-v2
25	10	10	GymnomeAssembly_25_10_10-v2
25	10	15	GymnomeAssembly_25_10_15-v2
50	10	8	GymnomeAssembly_50_10_8-v2
50	10	10	GymnomeAssembly_50_10_10-v2
50	10	15	GymnomeAssembly_50_10_15-v2
25	20	8	GymnomeAssembly_25_20_8-v2
25	20	10	GymnomeAssembly_25_20_10-v2
25	20	15	GymnomeAssembly_25_20_15-v2
50	20	8	GymnomeAssembly_50_20_8-v2
50	20	10	GymnomeAssembly_50_20_10-v2
50	20	15	GymnomeAssembly_50_20_15-v2
25	30	8	GymnomeAssembly_25_30_8-v2
25	30	10	GymnomeAssembly_25_30_10-v2
25	30	15	GymnomeAssembly_25_30_15-v2
50	30	8	GymnomeAssembly_50_30_8-v2
50	30	10	GymnomeAssembly_50_30_10-v2
50	30	15	GymnomeAssembly_50_30_15-v2
381	20	75	GymnomeAssembly_381_20_75-v2
567	30	75	GymnomeAssembly_567_30_75-v2
726	40	75	GymnomeAssembly_728_40_75-v2
930	50	75	GymnomeAssembly_930_50_75-v2
4,224	230	75	GymnomeAssembly_4224_230_75-v2

An environment for each dataset was created in the OpenAI Gym toolkit (Brockman et al., 2016) to share such RL challenges. These environments are available online (see Section 1 of the Supplementary Material (Padovani and Alves, et al., 2020)), where the reward system proposed in Approach 1.4 is used. The identification names of each environment are presented in the last column of Table 2. The seminal reward system is also implemented and available—version 1, which replaces v2 with v1 in the environment name field.

Two experiments were carried out to evaluate the approaches. In each experiment, 20 successive runs of each evaluated approach were performed for all 23 existing datasets (460 runs per approach). Given that each approach has different levels of complexity, the real execution time for each approach was considered for comparison. To reduce the interference of external factors in execution time, all experiments were individually and sequentially performed at the same station (with Ubuntu 16.04 in an AWS EC2 instance of the r5a.large type, dual core, 16 GB of RAM and 30 GB of storage).

In the first experiment (Experiment A), the objective was to verify the impact of progressively including new strategies. For this purpose, the performance of the seminal approach was evaluated (according to (Bocicor et al., 2011a)) against approaches 1.1, 1.2, 1.3, 1.4 (improved reward system), 2 (pruning dynamic) and 3.1 (genetic algorithm - GA). In the second experiment (Experiment B), the objective was to compare the performance of the new RL-based approaches against the performance of the GA alone. Therefore, in addition to Approaches 1.1, 1.2, 1.3, 1.4, 2 and 3.1, approach 3.2 (which explores the GA alone) was performed in an equivalent amount of time.

For the performance measure in each experiment, two percentage measures were calculated: the distance-based measure (DM) and the reward-based measure (RM). Evaluations of *de novo* assembly are typically performed using proper metrics, such as the N50 (Bradnam et al., 2013). These metrics were created because, as previously indicated, *de novo* assemblies are not supported by a reference genome. In some scenarios, it is not possible to assess the results obtained from the assemblers accurately because the optimal output is unknown. Here, although a *de novo* assembler is evaluated, its assessment environment is restricted, and the target genomes are known; this scenario allows the use of specific (and exact) evaluations, such as DM and RM metrics.

DM considers a successful run when the consensus sequence from the orders of the reads produced is identical to the expected sequence. RM considers any run as a success when the proposed order of reads represents the sum of PMnorm higher than or equal to the sum of PMnorm from the optimal read sequence (for details, see Section 3 of the Supplementary Material (Padovani and Alves, et al., 2020)).

## 3 Results

In Experiment A, the seminal approach consumed the longest running time (23 h and 34 min) and had the lowest average performance; an optimal response was obtained in 16.96% of the runs (i.e., 78 out of the 460 executions) in terms of distance from the expected genome (DM) and 21.30% (98 out of the 460) in terms of maximum reward (RM) (Table 3).

**TABLE 3:** Results of Experiment A. Comparison of the performances of trained agents with different reinforcement learning strategies. The performance of each approach is expressed using distance-based (DM) and reward-based (RM) metrics (see Methods for details).

Experiment a (approach)	Average DM	Average RM	Total Runtime
seminal	16.96%	21.30%	23 h34 m
1.1	9.57%	13.70%	19 h38 m
1.2	18.48%	21.30%	19 h38 m
1.3	20.00%	24.35%	19 h38 m
1.4	20.43%	24.78%	19 h38 m
2	20.65%	25.00%	18 h41 m
3.1	73.91%	80.87%	17 h03 m

Following the updated reward system, the DM and RM performances in Approaches 1.2, 1., 3, and 1.4 surpassed those of the previous approach and consumed approximately 4 h less (19 h and 38 min) of running time. Approach 3.1 presented the shortest running time, with a DM average of approximately 74% and an RM average above 80%, with the highest performance.

In Experiment B, approach 3.2 presented the shortest running time, with a DM average of 87% and an RM average of 95%. Given the superior performance of Approach 3.2, Experiment B applied the time taken by the genetic algorithm as a reference to find an optimal solution in terms of the RM for 22 out of the 23 datasets used (i.e., 95.65%), which corresponded to 1 h and 34 min of running time. Given the dominance of Approach 3.2, we also verified the performance of this approach on only the dataset with no optimal response (reads with 4Kbp). In this experiment, the running time for Approach 3.2 was considerably increased, lasting approximately 38 h (against less than 2 min for the same dataset for approach 3.2 in Experiment B, Table 4). No optimal solution was obtained for this dataset; however, it is possible to observe a consistent gain in performance in terms of both DM (where longer runs had shorter distances than most distances reached by shorter runs) and RM (which had higher accumulated rewards in all longer runs).

**TABLE 4:** Results of Experiment B. Experimental performance considering similar running times (RTs). The performance was expressed using a Distance-based Measure (DM) and Reward-based Measure (RM) (see Methods).

Experiment B (approach)	Average DM	Average RM	Total Runtime
1.4	13.91%	17.61%	01 h36 m
2	12.39%	16.30%	01 h36 m
3.1	14.78%	14.78%	01 h42 m
3.2	87.83%	95.65%	01 h34 m

## 4 Discussion

Genome assembly is among the most complex problems confronted by computer scientists within the context of genomics projects. When machine learning is applied to genome assembly, this complexity allocates the problem of finding optimal permutations of sequenced reads and reaching the target genome into an NP-hard problem, which comprises the most difficult problems in computer science (Roughgarden, 2020). This high complexity is particularly expressed in the vast state space required for representing the assembly problem in RL models.

In the approach studied here, according to Equation 1, reaching the optimal solution for sets of 30 reads requires the RL agent to explore a state space of approximately 2e44 states (Bocicor et al., 2011a) (2.10^44, which is more than the stars in the universe). In real-world scenarios, genomes are much larger. Applying RL combined with heuristics is a strategy for addressing complex problems, aiming at mapping actions into states that tend to maximize their reward, thus decreasing the computational complexity of the problem.

We aimed to expand the agent learning based on two constraints from the seminal approach for applying RL to the genome assembly problem: (1) the reward system and (2) the agent’s exploration strategies. We found that both improving the agent’s learning performance and updating the reward systems favored the agent to improve learning. Despite improvements, the system still occasionally produces suboptimal solutions. This is also supported by the fact that RM percentages were higher than DM percentages in some experiments.

The dynamic pruning mechanism showed slight improvement, but the additional processing cost and the benefit from its implementation did not indicate a reasonable net gain from its use as bypass for the problem emerging from the high dimensionality of the state space. Some of the gains were due to the improved agent’s performance, where the sum of rewards for the optimal permutation of reads was not maximized in the previous reward system. Despite the gains from the updated reward system, the inconsistencies were not completely resolved. In some of the datasets, the agent reached and even surpassed the maximum expected accumulated rewards without obtaining the target genome. A minor improvement is observed in approach 2, requiring approximately 1 h less processing.

The hybrid approach combining the RL strategy with a GA (Approaches 3) presented better performance. This combination was proven to be advantageous, probably given the curse of dimensionality encountered by the Q-learning algorithm, as strong GA support was observed for the agent while conducting the RL exploration. Despite these improvements, the approaches are not yet suitable for real-world scenarios. This is evident in the experiments performed with the largest dataset. Even the smallest genomes found in living organisms are larger than the largest dataset used in this study.

Nevertheless, none of the proposed approaches yielded an optimal solution for this dataset—not even the most effective one (GA)—when the execution time was extended. The superiority of the GA alone allows us to draw conclusions on the current infeasibility of applying the Q-learning algorithm to solve the genome assembly problem in search of an optimal read permutation, as proposed in the seminal approach.

Given the absence of approaches in the literature for tackling this problem through RL and considering the optimistic results obtained by RL in other areas (especially when RL is combined with deep learning) (Mnih et al., 2015), further investigations on the applicability of RL, including the use of different modeling approaches and algorithms, are needed.

One of the major challenges in applying RL to real-world problems is the low sample efficiency of the algorithms (Yu, 2018). Considering the time required by the agent trained by the Q-learning algorithm to reach an optimal solution, it is possible to perceive a high need for numerous interactions with the data. Considering that genome inputs are larger than those experimentally applied here, obtaining a sample efficient algorithm for the problem is at the core of developing a real-world solution. Additionally, the agent sample efficiency must be optimized to explore the state space, which might be achieved by the application of techniques to remove duplicate reads—due to repeats—and the use of an intrinsic motivation to bypass the exploration problem, given the high dimensionality of the proposed state space (Yu, 2018; Barto, 2012).

Future research should also focus on exploring and systematically comparing different reinforcement learning algorithms for the genome assembly problem (Yassine and Riffi, 2023). While this study focused on Q-learning, other approaches—such as policy gradient methods, actor‒critic algorithms, and additional reinforcement learning techniques—may offer more suitable mechanisms for capturing the sequential decision-making and structural complexity involved in the task (Shakya et al., 2023; Grondman et al., 2012). A comparative analysis of these algorithms could provide valuable insights into their effectiveness, limitations, and applicability, ultimately guiding the development of more robust and scalable solutions in this domain.

The use of graph embedding may act as another option for modeling approaches allowing the use of deep RL without requiring the conversion of the problem into an image—the genome assembly problem may be represented through a graph, in the shape of the traveling salesman problem (TSP) (Cook, 2012; Li et al., 2011).

As highlighted throughout this study, the limitations observed in the application of Q-learning to the fragment assembly problem suggest that traditional reinforcement learning techniques may not be sufficient to handle the complexity and scalability required in real-world scenarios. Therefore, a fundamental direction for future research is the exploration of deep reinforcement learning (DRL) techniques (Osborne et al., 2022). DRL has the potential to address the high-dimensional state and action spaces inherent to the assembly problem, enabling more robust generalization and improved decision-making (Mnih et al., 2015; Hafner et al., 2025).

Another key direction for future research is the exploration of transfer learning in the context of RL-based genome assembly. Leveraging transfer learning techniques could enable the development of more practical and robust assembly models that generalize across different datasets, reducing the need for retraining from scratch for each new scenario (Taylor and Stone, 2009; Yang et al., 2020; Zhu et al., 2023). By allowing previously acquired knowledge to inform new learning tasks, transfer learning has the potential to significantly increase the efficiency and scalability of RL-based genome assemblers, paving the way for broader applicability and real-world use.

Finally, one last aspect to be considered for the adoption of RL in the genome assembly problem is the generalization of the agent’s learning, which is a major challenge for the use of RL in real-world problems (Ponsen et al., 2010). As designed for the RL environment for the genome assembly problem, the learning acquired by the agent when assembling a set of reads will hardly be applied for the assembly of a new set.

Although the results obtained have shown that the application of Q-learning to genome assembly, as proposed in the seminal approach, does not yield satisfactory performance at larger scales, the main scientific contribution of this work lies in addressing a current gap in knowledge. To date, the only existing proposal in the literature has explored this approach using extremely small datasets without assessing its feasibility in more realistic scenarios. By conducting a broader analysis with relatively larger datasets and adaptations to the original algorithm, this study provides a critical and well-founded evaluation of the limitations of this technique. Thus, even though the results do not point to a promising solution, they advance scientific understanding of the subject by more clearly delineating the challenges and constraints involved in applying reinforcement learning methods to genome assembly.

All the experiments and the RL environments used in this study are publicly available and open for reuse (for details, see Section 5 of the Supplementary Material (Padovani and Alves, et al., 2020)) to support future studies.

## 5 Conclusion

This study provides a comprehensive evaluation of the applicability of reinforcement learning (RL), specifically the Q-learning algorithm, to the genome assembly problem. While initial results using the seminal approach confirmed its functionality on small datasets, our expanded analyses revealed critical scalability limitations. Through a series of methodological improvements, including the revised reward systems, dynamic pruning, and the incorporation of evolutionary algorithm (Genetic Algorithm–GA), we demonstrated incremental performance gains. However, even the most advanced hybrid strategies failed to deliver optimal results on larger, more realistic datasets. Notably, the genetic algorithm alone outperformed all RL-based strategies, highlighting the current inadequacy of Q-learning for addressing the high-dimensional state spaces inherent to genome assembly. These findings underscore the importance of exploring alternative RL algorithms, such as deep reinforcement learning and policy gradient methods, alongside strategies like transfer learning and intrinsic motivation. Despite the lack of a viable RL-based solution at present, this study contributes with a valuable benchmark for future research by mapping the limitations of current approaches and emphasizing key directions for advancing machine learning applications in genome assembly.

## Data Availability

The original contributions presented in the study are included in the article/supplementary material, further inquiries can be directed to the corresponding author/s.
