# Supplementary material for: Using reinforcement learning in genome assembly: in-depth analysis of a Q-learning assembler
Source: Front Bioinform. 2025 Aug 20;5:1633623. doi: 10.3389/fbinf.2025.1633623 (PMC12405310; doi:10.3389/fbinf.2025.1633623)
Supplement: Supplementary file 1 [file DataSheet1.pdf]

Supplementary Material (Using reinforcement learning in genome assembly: in-depth analysis of a Q-learning assembler)

1 Section 1 — Genome assembly environments for OpenAI Gym

In this section, the reinforcement learning environments will be presented, built following the guidelines from the well established toolkit for developing and comparing reinforcement learning algorithms, OpenAI Gym. The purpose of creating and providing these environments is to expand the range of existing reinforcement learning tasks in the scientific community considering real world problems — in our case study, the assembly of genomes.

We provide 46 reinforcement learning environments related to genome assembly, all publicly available at `https://bit.ly/3YWFtkm`. The descriptions (e.g. the states space, actions space and rewards systems) for the 23 environments used for conducting the experiments of the study, hence forward referred to as main text, to which this supplementary material belongs, are provided.

The environments were separated into two versions, *v1* and *v2*, differentiated only by the rewards system applied. The environments of version v1 apply the rewards system proposed in Bocicor et. al [1]. In version v2, the reward system corresponds to the approach 1.4 proposed in the main text.

Both versions share the same states space, which represents all possible arrangements for the *reads* being assembled. In environments of v1, every action leading the agent to a state representing an arrangement that is a non-repetition permutation produces a reward that corresponds to the sum of a constant and the overlap found between consecutive pairs of *reads* and low value reward for the remaining actions.

In version v2, this constant reward applies only to actions leading to states representing arrangements with a single read; all remaining actions produce rewards corresponding to the overlap (normalized, between 0 and 1) between the last two reads in the arrangement and, additionally, a constant value of 1.0 is added to those leading to states that represent non-repetition permutations.

The actions space, in both versions, is composed by one action for each *read* assembled. Therefore, if the dataset contains n *reads* for being assembled, the agent will have n available actions in each state. For each environment a fixed integer value m was identified for each *read*, such that $0 \leq m \leq n-1$ and, with that, each action is represented by an integer number (i.e. $Discrete(n)$).

Following the OpenAI Gym specifications, each state of the states space is represented by an array consisting of n integers (i.e. $Box(n, dtype = numpy.int32)$), where n corresponds to the number of *reads* to be assembled in the set. This array will correspond to the arrangement of used *reads*, with the element -1 indicating the end of the arrangement. Thus, the initial state (which, as seen, is unique), is represented by an array exclusively composed by -1.

Both versions are designed for the episodic reinforcement learning. Every episode starts with the initial state and is interrupted every time an absorbing state is reached (regardless of that state is corresponding or not to a non-repetition permutation).

These 46 environments were built based on 23 sets of distinct *reads* and obtained from the sequencing simulation method using different microgenomes. For each set of *reads*, two versions were provided. The name of each environment is defined by a text identifying the set of *reads* followed by the suffix *-v1* or *-v2*, according to the user´s requirement. The identifiers of each environment can be found in the environments list, provided in the environments tutorial, available online[1].

In addition to the two versions, these environments are different in 3 other aspects: (a) size of the microgenome used in the simulation; (b) number of *reads* to be assembled; and (c) number of characters for each read in the set. Both the produced *reads* and the microgenomes used for producing them may be accessed through the environment, as presented in the usage tutorial.

2 Section 2 — Normalized overlap calculation

In this section, an example of the normalized overlap calculation between *reads* will be presented, referred to in the main text by $ol_{norm}(s, s')$. In this notation, s indicates the state prior to the action made to reach s'. Thus, here we want to calculate the normalized overlap resulting from the agent´s action leading from s to s'.

In the example, let's consider three reads, referred to as A, B and C, so that $read_A = AAACC$, $read_B = ACCCG$, and $read_C = CGTTT$. We will also consider 4 states s_W, s_X, s_Y, and s_Z, so that s_W corresponds to the initial state; s_X corresponds to the state reached by s_W when the action corresponding to the $read_A$ is taken; s_Y is the state reached by s_X when the action corresponding to the $read_B$ is taken; and s_Z corresponds to the state reached when the action corresponding to the $read_C$ is taken while in s_Y (i.e., $W \rightarrow^A X \rightarrow^B Y \rightarrow^C Z$).

As previously stated, in the overlap calculation (unlike what was proposed in [1]), the relative orientation of the pair of *reads* under analysis was considered

[1]The URL where the environments are available is shown in the main text

for identifying the overlap. For example, when calculating the exact overlap (before normalizing) between $read_A \rightarrow read_B$ a suffix-prefix overlap of 3 characters (corresponding to ACC) was found, but when calculating the overlap $read_B \rightarrow read_A$, we found an exact suffix-prefix overlap that corresponds to 0. The same is applied when calculating for the *reads* B and C which have a overlap equals to 2 when $read_B \rightarrow read_C$ (referring to CG) and equals to zero in the reverse orientation.

Therefore, in our example, when calculating $ol_{norm}(s_X, s_Y)$, we should consider the overlap $read_A \rightarrow read_B$, as s_X was achieved when taking the action referred to the $read_A$ and s_Y when taking the action referred to the $read_B$. Similarly, to calculate $ol_{norm}(s_Y, s_Z)$ we should consider the overlap $read_B \rightarrow read_C$. Here, the notation $read(s)$ is used for referring to the read whose action allowed to achieved s (i.e. $read(s_X) = read_A$, $read(s_Y) = read_B$, and so on).

For the calculating $ol_{norm}(s, s')$, we must first calculate the exact suffix-prefix overlap between $read(s) \rightarrow read(s')$, called $ol(s, s')$; this overlap is then normalized, so that it ranges between 0 and 1, considering the rate of this overlap in relation to the product between the number of *reads* in the set (n) and the size of the biggest *read* (m). The Equation 1 defines $ol_{norm}(s, s')$ mathematically.

$$ol_{norm}(s, s') = \frac{ol(s, s')}{n \times m} \tag{1}$$

In our example, we have that non-normalized overlaps $read_A \rightarrow read_B$ and $read_B \rightarrow read_C$ are equal to 3 and 2, respectively, and it is known that $n = 3$ and $m = 5$, as there are 3 *reads* with 5 characters each[2]. Based on this, we can conclude that $ol_{norm}(s_X, s_Y) = 0.2$ (given that $\frac{ol(s_X, s_Y)=3}{3 \times 5} = 0.2$); we can also conclude that $ol_{norm}(s_Y, s_Z) \approx 0.13$ (given that $\frac{ol(s_Y, s_Z)=2}{3 \times 5} = 0.33...$).

This normalization allows that, mathematically, the sum of overlaps between all *reads* in a permutation without repetition would never reach 1.0. This follows the subjacent logic that the highest sum of overlaps between n *reads* will occur when all *reads* are completely superimposed. This is because all *reads* present exactly the same content; and, consequently, the overlap between each pair of *reads* will always be equal to m, being m the number of characters in the *reads*. Thus, knowing that there are $n - 1$ consecutive pairs of reads in a permutation of n *reads*.

In this extreme case, the non-normalized overlap between any consecutive pair among n *reads* will correspond to m — once that all have the same m number of characters. Therefore, the normalized overlap for any pair of *reads* will be equivalent to $\frac{m}{n \times m} = \frac{1}{n}$. The sum of the normalized overlap of all $n - 1$ consecutive pairs, according to the mathematical property of constant summation corresponds to $1 - \frac{1}{n}$. As $n \in \mathbb{Z}^+$, it may be concluded that $0 < \frac{1}{n} \leq 1.0$, consequently, we conclude that $0 \leq 1 - \frac{1}{n} < 1.0$. This allows us to conclude that the sums of the normalized overlaps will range between $[0, 1)$ for all cases.

[2]Here, the number of characters of each *read* is the same for all of them, however, in real scenarios, it might have variation and m must be set to the greatest read size in such case.

As stated in the main text, this normalized overlap measure was applied given the new rewards system proposed, but it was also applied for calculating the fitness function of each individual in approach 3 (which uses the genetic algorithm). The details for calculating the objective function will be presented in Section 4 of this supplementary material.

3 Section 3 — Performance measures for the intelligent agents

In this section, we present the details for calculating the two performance measures applied in the main text for measuring the performance of intelligent agents during the task of assembling the proposed sets of *reads*: the Distance Measure (DM) and the Reward Measure (RM).

Two performance metrics were applied because they measure the learning from different perspectives. The DM evaluates the agent's performance considering the assembly task that must be solved (i.e. it evaluates the optimality of the results achieved in terms of the achieved genome). In the RM, considering that the agent's generic objective is to maximize the accumulated rewards received, it evaluates the agent's learning in a generic matter, regardless of the task - that is, it assesses the agent's ability to obtain higher accumulated rewards. Thus, it is possible to assess whether there is a sample efficiency problem and/or the inadequacy of the rewards system.

As mentioned in the main text, given the implicit randomness in the agents´ training, the performances were estimated based on a number of different runs, in order to assess the results consistency after many runs. This way, in both metrics, a favorable or unfavorable result is obtained at the end of each run. For example, in the DM case, if at the end of 20 runs the agent was able of reaching the target genome in only 10 of those runs, it is assumed that its DM performance is 50%.

For assessing whether a given run was positive regarding PM results, the expected value of accumulated rewards for optimal permutations in each dataset was considered - that is, *reads* were aligned alongside the known microgenomes and for each data set, the accumulated rewards values for optimal permutations were obtained, thus reaching the expected performance.

As the generic challenge of reinforcement learning agents trained by the Q-learning algorithm is to maximize accumulated rewards, generically the goals were achieved by the agents when, at least, the desired accumulated reward value was reached. Thus, a run is positive if the sum of rewards is equal or higher than the expected value.

As previously indicated, when considering DM, a run is positive when the genome assembled by the agent corresponds exactly to the known target microgenome. For this evaluation, two steps are performed: (1) achieving an assembled genome and (2) comparing the achieved genome to the target microgenome.

The achieved genome corresponds to the consensus sequence obtained by combining pairs of *reads* in the order designated by the agent´s permutations. For example, if the agent replies, in the end of the training, that the permutation achieved is formed by *reads* B, C and A, in this order, the genome achieved by that agent will correspond to the characters' sequence composed by the content of those *reads* in that order, integrating the overlap between pair. Figure 1 presents an example of a hypothetical content for each of these *reads* and achieve a corresponding consensus sequence.

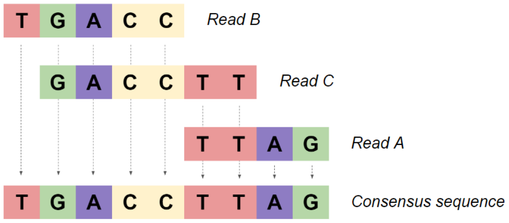

Figure 1: Example for obtaining an exact consensus sequence from a permutation composed, in this order, by *reads* B, C and A, being these *reads* composed by the sequences *a*.

Finally, the genome achieved by the agent is compared to the microgenome (known) that generated the corresponding *reads*. This comparison is measured by the *Levenshtein* distance [3]. This distance briefly reflects the minimum number of insertions, deletions and/or character changes that must be made in one of the two texts so that both become exactly the same (for example, the distance between 'book' and 'back' is equal to 2, as two changes must be made to transform 'book' into 'back', or vice versa). Therefore, DM consider as positive any run that obtained a genome that presents the distance from the target microgenome equal to zero.

4 Section 4 — Specificities of the evolutionary computing algorithm

As presented in the main text, approach 3 uses evolutionary computation by applying the genetic algorithm. The genetic algorithm was implemented with the elitist selection of individuals - thus, the most fit individual in the population reached by the $i-th$ generation is automatically taken for the generation $i+1$.

The number of individuals in the population was preset and the chromosome of each individual was composed of n genes, so that n corresponds to the number of *reads* in the set for being assembled. The allele of each gene corresponds to the *read* identification, without the possibility of repeating alleles. Therefore, each chromosome can be viewed as a simple permutation of n *read*.

The fitness function calculation for each individual corresponds to the sum

of the normalized overlaps between all pairs of *reads* in consecutive genes from the corresponding chromosome. For additional details on the calculation of the normalized overlap, see Section 2 of this material.

From each new generation, three stages are implemented: elitism, crossover and mutation. With the exception of the initial population, elitism occurs in all populations, the most fit individual from the previous population is cloned into the current population. Then, crossover (for generating two new individuals) and mutation (which may change the genetic code of the new individuals), which will be described below.

The crossover operation initially selects random pairs of parents, however prioritizing the best adapted parents. Each cross between parents produces two offspring, which, according to previously defined probabilities, may be cloned offspring from their parents - one children for each - or offsprings whose chromosomes are composed by the combination of genes from each of the parents - at random from ensuring that alleles are not repeated.

Subsequently, each offspring may have two of its genes mutated (following predefined probabilities) in order to change the alleles of those genes in the chromosome - thus maintaining non-repeated alleles. The actions of elitism, crossover and mutation occur continuously until the offspring number from the new generation reaches the number of individuals expected for the population.

5 Section 5 — Experiments reproducibility

In this section, a technical guideline for repeating the experiments referred to in the main text is presented. As mentioned, two experiments were carried out, called *Experiment A* and *Experiment B* – four rounds of tests were carried out (testing the approaches 1, 2 and 3.1 in both experiments and, additionally, the seminal approach and the approach 3.2, in the Experiments A and B respectively). Finally, the approach 3.2 was extrapolated for the larger dataset. Table 1 presents a summary of the tests performed and an identification code (test id) was assigned for each test, which later will be used for the instructions of repeatability.

These experiments were organized in folders in the file system, the names correspond to the Test id provided in Table 1 and are compressed into a single file (*reproduce.zip*) obtained from the data repository and available `https://osf.io/tp4zj/files/`.

All experiments, as described in the main text, were performed in an environment with Linux operating system (Ubuntu 16.04). for repeating the experiments, it is necessary to install the *Python 3+* software and the Python *Numpy 1.18.5+* module. After installing all dependencies, each experiment can be reproduced by executing the executable file *reproduce.sh*, located inside the folder of each experiment.

Figure 2 presents a sample script for installing the dependencies and reproducing the experiments in the *Docker* platform. This example considers that the aforementioned compressed file is stored in the current folder and the folder

Description	Test id
Seminal Approach tested in Experiment A	*experimentA_Seminal*
Approach 1.1 tested in Experiment A	*experimentA_Approach1.1*
Approach 1.2 tested in Experiment A	*experimentA_Approach1.2*
Approach 1.3 tested in Experiment A	*experimentA_Approach1.3*
Approach 1.4 tested in Experiment A	*experimentA_Approach1.4*
Approach 2 tested in Experiment A	*experimentA_Approach2*
Approach 3.1 tested in Experiment A	*experimentA_Approach3.1*
Approach 1.4 tested in Experiment B	*experimentB_Approach1.4*
Approach 2 tested in Experiment B	*experimentB_Approach2*
Approach 3.1 tested in Experiment B	*experimentB_Approach3.1*
Approach 3.2 tested in Experiment B	*experimentB_Approach3.2*
Approach 3.2 tested in superior time B	*experimentC*

Table 1: List of experiments performed.

name, highlighted in gray, must be replaced by the respective experiment identification. Excepting the first line of the code, all remaining lines are executable on Linux operating systems regardless of the previous installation of the Docker platform.

In summary, the steps to reproduce the experiments are given below. Figure 2 shows an example of execution using Docker.

1. Download and extract the supplementary file (reproduce.zip)
2. Install Python
3. Install Numpy package
4. Access the folder corresponding to the approach you wish to reproduce
5. Execute reproduce.sh

6 Section 6 — Seminal reward system inconsistency

The reward system used for training RF agents ideally should somehow correlate high quality responses with high rewards — what is not always true for our seminal work. In Figure 3 we can observe such inconsistency through an example, which presents two different permutations for a set of *reads* obtained from a given (and known) microgenome — identified by letters ranging from A to J. The first permutation is formed, in this order, by the *reads* A, B, C, D, E, F, G, H, I, and J, and, according to SW algorithm, the accumulated overlap (aka PM) is 40.34. Such score was obtained by summing the maximum overlap, calculated by SM algorithm, between *reads* A and B with the maximum overlap

```
docker run -it ubuntu:16.04
apt update
apt install -y python3
apt install -y unzip
apt install -y curl
curl https://bootstrap.pypa.io/get-pip.py -o get-pip.py
python3 get-pip.py
pip3 install numpy
unzip reproduce.zip
cd <experimentID>
./reproduce.sh
```

Figure 2: Sample script to run each of the aforementioned experiments.

between *reads* B and C, and so on. Table 2 presents the maximum overlap between all pairs of subsequent *reads* in this permutation.

**A-B**	**B-C**	**C-D**	**D-E**	**E-F**	**F-G**	**G-H**	**H-I**	**I-J**
4	5	4	5	5	3.67	4	2.67	7

Table 2: Maximum overlap between each pair of subsequent *reads* of the permutation A-B-C-D-E-F-G-H-I-J, obtained by using the SW algorithm (using $match = 1.0$, $mismatch = -0.33$, and $gap = -1.33$), thus producing a cumulative overlap of 40.34.

The aforementioned permutation (from A to J) corresponds to the optimal permutation, because the union of subsequent *reads* through the maximum exact overlap between the suffix of the previous read and the prefix of the next read produces exactly the target microgenome (provided at the bottom of the figure as the output genome).

We can see in Table 2 that the overlap between *reads* I and J is equal to 7, and it is easy to understand such value when we observe the alignment of the corresponding *reads* in Figure 3 — we have 7 letters matching between the sequences (i.e., GACACCC). However, it is not that trivial to understand why the SW algorithm reached a maximum overlap of 4 letters between *reads* A and B — since the way they are aligned in Figure 3 suggests only 1 overlapping letter (i.e., A). We will return to this case and explain it further later.

Similarly, we can analyze the second permutation, formed by the *reads* H, G, F, E, C, B, D, A, J, and I, respectively, is not optimal, as the corresponding output genome (obtained using the same read union procedure mentioned above) is quite different (and also longer) from the target genome. However, for this permutation, the SW algorithm presents an accumulated overlap of 43.02, which is greater than that obtained for the optimal permutation and can be achieved by summing up the overlaps between each pair of subsequent *reads*, presented in Table 3.

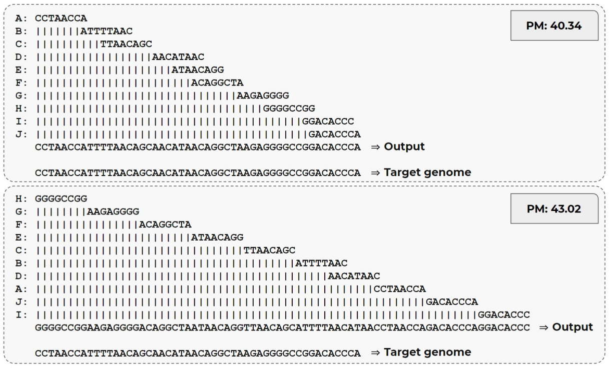

Figure 3: Illustration showing that the measure used as a reward to train agents does not produce maximum values for optimal outputs in some cases. Above, we have an optimal permutation of *reads*, for which the PM is 40.34 and whose corresponding genome is equal to the target genome itself; and, below, we have another permutation whose output differs from the target genome, but the corresponding PM is greater than the PM of the optimal permutation.

A-B	B-C	C-D	D-E	E-F	F-G	G-H	H-I	I-J
4	3.67	5	6	5	4.01	4.67	3.67	7

Table 3: Maximum overlap between each pair of subsequent *reads* of the permutation H-G-F-E-C-B-D-A-J-I, obtained by using the SW algorithm (using $match = 1.0$, $mismatch = -0.33$, and $gap = -1.33$), thus producing a cumulative overlap of 43.02.

We can see in Table 3 that the overlapping score calculated by SW algorithm considering *reads* I and J is 7; but, again, it may be not clear to understand it, because the way they are aligned in Figure 3 suggests no overlapping between them. The reason SW algorithm produces an overlapping score equals to 7 is that such algorithm does not constraint an order between the pair of sequences.

```
A:   CCTAACCA
B: ATTTTAAC
```

Figure 4: Illustration to emphasize that the overlap score calculated by SW algorithm does not take in to account the relative order of the pair of sequences.

In that case, it will return the maximum overlap, whether obtained considering *read* I to the left of read J, or vice versa — and, as we mentioned above, in the first permutation, we have a 7-letter overlap when considering that *read* I is to the left of *read* J. This is the same reason why we have, even in the first permutation, a 4-letter overlap between *reads* A and B, because, considering

read B is to the left of A (and not the opposite), it is possible to achieve a 4-letter overlap between the sequences (i.e., TAAC), as illustrated in Figure 4.

7 Section 7 — Literature review

The literature review of this work was conducted using the following search string: *"reinforcement learning" AND assembl* AND (*genome* OR *omics OR bioinformatic* OR dna OR rna OR genetic* OR contig* OR sequencing OR scaffolding OR "de novo" OR ngs).* Only documents written in English were considered, while publications from fields unrelated to genome assembly (described in Table 4), conference reviews, and works not directly addressing the genome assembly problem were excluded. This review resulted in 3 papers[1, 2, 4].

**Area**
Automation Control Systems
Business, Management and Accounting/Business, Management and Accounting
Business Economics
Construction Building Technology
Decision Sciences
Earth and Planetary Sciences
Energy
Geography
Imaging Science Photographic Technology
Instruments instrumentation
Materials Science
Mathematics
Mechanics
Neuroscience
Nuclear Science Technology
Operations Research Management Science
Physics/Physics and Astronomy
Psychology
Robotics
Telecommunications
Transportation

Table 4: List of excluded areas from the review

References

[1] Maria-Iuliana Bocicor, Gabriela Czibula, and Istvan-Gergely Czibula. A reinforcement learning approach for solving the fragment assembly problem.

In *2011 13th International Symposium on Symbolic and Numeric Algorithms for Scientific Computing*. IEEE, September 2011.

[2] Maria-Iuliana BOCICOR, Gabriela CZIBULA, and István Gergely CZIBULA. A distributed q-learning approach to fragment assembly. *Studies in Informatics and Control*, 20(3), September 2011.

[3] Vladimir Iosifovich Levenshtein. Binary codes capable of correcting deletions, insertions and reversals. *Soviet Physics Doklady*, 10(8):707–710, 1966. Doklady Akademii Nauk SSSR, V163 No4 845-848 1965.

[4] Roberto Xavier, Kleber Padovani de Souza, Annie Chateau, and Ronnie Alves. *Genome Assembly Using Reinforcement Learning*, page 16–28. Springer International Publishing, 2020.
